# Characterization of QTLs and Candidate Genes for Days to Heading in Rice Recombinant Inbred Lines

**DOI:** 10.3390/genes11090957

**Published:** 2020-08-19

**Authors:** Youngjun Mo, Jong-Min Jeong, Su-Kyung Ha, Jinhee Kim, Changmin Lee, Gung Pyo Lee, Ji-Ung Jeung

**Affiliations:** 1 National Institute of Crop Science, Rural Development Administration, Wanju 55365, Korea; moyj82@korea.kr (Y.M.); jjm0820@korea.kr (J.-M.J.); rocksue193@korea.kr (S.-K.H.); jinhee2723@korea.kr (J.K.); cropas@korea.kr (C.L.); 2 Department of Integrative Plant Science, Chung-Ang University, Anseong 17546, Korea; gplee@cau.ac.kr

**Keywords:** rice, days to heading, QTL, *Hd1*, *Ghd7*, *Hd16*

## Abstract

Understanding the gene mechanisms controlling days to heading (DH) is important in rice breeding for adaption in the target environment. Using a recombinant inbred line population derived from the cross between two *japonica* rice cultivars, Koshihikari and Baegilmi, we identified three consistent quantitative trait loci (QTLs) for DH for two years, *qDH3*, *qDH6*, and *qDH7* on chromosomes 3, 6, and 7, respectively. While Baegilmi contributed the allele for early heading at *qDH6* and *qDH7* with the additive effect of five days each, Koshihikari contributed the allele for early heading at *qDH3* with the additive effect of three days. Notably, pyramiding two or more alleles for early heading at these QTLs accelerated heading effectively. Sequencing of *Hd16*, *Hd1*, and *Ghd7*, the previously known heading date genes underlying *qDH3*, *qDH6*, and *qDH7*, respectively, revealed that Baegilmi and Koshihikari carry different alleles at the three genes. Molecular markers were developed to screen the allelic compositions of the three genes among 295 Korean commercial rice cultivars. The results showed that few cultivars carry alleles for early heading at the three genes, highlighting that DH can be further accelerated and fine-tuned in breeding programs by combining the desirable alleles of *Hd16*, *Hd1*, and *Ghd7*.

## 1. Introduction

The transition from vegetative to reproductive stage is a critical developmental event in plants for ensuring offspring survival under favorable environments [1]. Understanding the genetic basis of flowering time control is especially important in crop species for breeding cultivars adapted well in the target environment. In rice, flowering (also referred to as heading) time is regulated by the complex genetic mechanisms involving hundreds of quantitative trait loci (QTLs) and at least 14 cloned genes [2,3].

Various environmental cues are integrated to modulate the expression of genes encoding florigen, which is synthesized in leaves and transferred to the apical meristem to initiate flower development [4]. In rice, florigen is encoded by *Heading Date 3a* (*Hd3a*) and *RICE FLOWERING LOCUS T 1* (*RFT1*), the orthologs of Arabidopsis *FT* [5,6]. *Hd3a* expression is mainly regulated by *Hd1*, the ortholog of Arabidopsis *CONSTANS* (*CO*), which upregulates *Hd3a* under short day and downregulates *Hd3a* under long day [7]. *Hd1* expression is upregulated under both short and long days by *OsGI*, the ortholog of Arabidopsis *GIGANTEA* (*GI*) [8]. Unlike bifunctional *Hd1*, *EARLY HEADING DATE 1* (*Ehd1*) can accelerate heading under both short and long days by upregulating *Hd3a* and *RFT1* under short and long days, respectively [9]. *Ehd1* expression is negatively regulated by *Grain number*, *plant height*, *and heading date 7* (*Ghd7*) under long day [10]. While the *OsGI*-*Hd1*-*Hd3a* pathway in rice is orthologous to the *GI*-*CO*-*FT* pathway in Arabidopsis, the *Ghd7*-*Ehd1*-*Hd3a*/*RFT1* pathway is unique in rice without clear orthologs in Arabidopsis [1]. Recent studies revealed more complex rice-specific gene networks regulating the *Ghd7*-*Ehd1*-*Hd3a*/*RFT1* pathway (e.g., *Hd16* and *Hd17* upregulating *Ghd7* under long day) reviewed in [2,3].

To fine-tune days to heading (DH) in breeding programs and maximize yield and grain quality under the target environment, it is essential to characterize the effects of major genes controlling DH, their allelic variation, epistasis, and interaction with environmental factors including daylength and temperature. In Korea, developing early heading rice cultivars is especially important for boosting farmers’ income by enabling diverse double cropping patterns in the rice paddies, e.g., late-planting of rice after harvesting winter crops such as cabbage, barley, and wheat, or early-planting of rice followed by cash crops such as garlic and onion [11,12]. The utilization of early heading rice cultivars can be also useful for reducing cropping duration in order to minimize damages from erratic weather events. Although over 80 rice cultivars classified as the early heading group have been released in Korea, they occupy less than 10% of the rice cultivation area in Korea as many rice growers generally prefer mid-late heading cultivars because of their higher yield and grain quality compared to the early heading cultivars [13].

Baegilmi is an extremely early heading rice cultivar recently released in Korea exhibiting high yield in the mid-north plain (milled rice yield 5.01 MT/ha) and north-east coastal (5.27 MT/ha) regions in Korea [14]. However, the genes conferring early heading in Baegilmi have remained unknown. In this study, we used a recombinant inbred line (RIL) population derived from the cross between Koshihikari and Baegilmi to identify the chromosomal regions harboring genes controlling DH. Candidate genes underlying the major DH QTLs were sequenced in Koshihikari and Baegilmi and their allelic compositions were screened among commercial rice cultivars. Molecular breeding strategy using the allelic variations in major DH genes was discussed to facilitate the fine-tuning of DH in breeding programs.

## 2. Materials and Methods

### 2.1. Plant Materials and Phenotype Evaluation

Two *japonica* rice cultivars, Koshihikari with high eating quality [15] and Baegilmi with early maturity [14], were used in this study. Days to heading (DH) and grain filling rates of the two cultivars were evaluated at the experimental field of the National Institute of Crop Science (NICS), Suwon, Korea (37°27′ N 126°99′ E) in 2014. The seeds of each cultivar were sown on 25 April and transplanted on 25 May under a randomized complete block design (RCBD) with three replications. Each plot comprised of eight 4.5 m rows, with 30 hills per row and three plants per hill. The hills within a row were spaced by 15 cm and the rows were spaced by 30 cm. DH was determined by counting the number of days from sowing to heading when the panicles emerged in 40% of the plants in a plot. To evaluate the rate of grain filling of Baegilmi and Koshihikari, changes in grain weight of the two cultivars were monitored by measuring 1000 grain weight every three to four days during 19–50 days after heading.

To map QTLs for DH, a RIL population (*n* = 142) was constructed from the cross between Koshihikari and Baegilmi by the single seed descent method. The RIL population (F_6_ and F_7_ generation in 2016 and 2017, respectively) and its parents were grown at the experimental field of NICS, Wanju, Korea (35°84′ N 127°05′ E) in 2016 and 2017. The seeds were sown on 9 May and 10 May in 2016 and 2017, respectively, and the seedlings were transplanted four weeks after sowing. Each RIL was transplanted in a 4.5 m row with the individual plants spaced by 15 cm (30 plants per each line) and the rows spaced by 30 cm. DH of each RIL was determined by counting the number of days from sowing to heading when the panicles emerged in 40% of the plants in a row.

Days to heading of 295 commercial rice cultivars released by NICS, Rural Development Administration, Korea, were evaluated under the optimum and early planting conditions at the experimental field of NICS, Wanju, Korea. Sowing and transplanting dates for optimum planting were 9 May and 1 Jun, respectively in 2018, and those for early planting were 10 Apr and 9 May, respectively in 2019. Planting density and DH evaluation were as described above for the RIL population.

### 2.2. Sequencing Library Construction and Genotyping

Genomic DNA was extracted from the fresh young leaves of the Koshihikari × Baegilmi RILs using the CTAB (cetyl trimethylammonium bromide) method [16] with minor modifications. The quality and quantity of the extracted DNA were checked using the DeNovix DS-11 spectrophotometer (DeNovix, Wilmington, DE, USA) and the Quant-iT^TM^ dsDNA assay kit (Thermo Fisher Scientific, Waltham, MA, USA). The genotyping-by-sequencing (GBS) library was constructed according to [17]. Briefly, each DNA sample was digested with the *ApeKI* restriction enzyme (New England Biolabs, Ipswitch, MA, USA), ligated with barcode adapters, pooled, and purified using the QIAquick PCR purification kit (Qiagen, Hilden, Germany). The pooled libraries were amplified by PCR with an adapter-specific primer set, analyzed for the target length of 170–350 bp using BioAnalyzer 2100 (Agilent, Santa Clara, CA, USA), and sequenced using the Illumina HiSeq 2000 platform (Illumina, San Diego, CA, USA). The sequencing reads were mapped to the Nipponbare IRGSP-1.0 reference using the BWA aligner [18] and single nucleotide polymorphism (SNPs) were extracted in the format of a VCF (variant calling format) file. The SNP filtering and genotype calling of each RIL were carried out using TASSEL-GBS v2 [19] with the filtering conditions (mapping quality ≥30, base quality ≥20, and coverage ≥7) as described in [20]. Genotype calling for each SNP was conducted by defining a homozygous genotype when the same sequence rate is over 0.90 and a heterozygous genotype when the alternative sequence rate is 0.25–0.27.

### 2.3. Linkage Mapping and Statistical Analysis

Of 893 SNPs segregating in the RIL population, 128 high quality SNPs were selected for the linkage map construction after excluding SNPs with missing rate over 10%, significant segregation distortion (Chi-square test *p*–value < 0.05), or overlapping genetic positions. The composite interval mapping was implemented using QTL IciMapping version 4.1 [21] with the threshold LOD (logarithm of the odds) score of 3.0. The same program was also used to estimate the additive effects and the phenotypic variation explained by each QTL at the peak LOD. The mean comparisons of DH between the RILs with different allelic combinations of the identified QTLs were conducted by the Duncan’s multiple range test using SAS version 9.4 (Cary, NC, USA). Three-way factorial ANOVAs were conducted to study the main effects of the three DH QTLs and their two-way and three-way interactions using SAS version 9.4.

### 2.4. Sequencing and Marker Analysis for Hd16, Hd1, and Ghd7

The coding regions of *Hd16*, *Hd1*, and *Ghd7*, the candidate genes underlying the three DH QTLs from the Koshihikari × Baegilmi RIL population, were sequenced to search polymorphisms between Koshihikari and Baegilmi. The tenth exon of *Hd16* was amplified by polymerase chain reaction (PCR) using the primers and conditions described in Supplementary Table S1. The two exons of *Hd1* and the two exons of *Ghd7* were amplified by PCR as described in [22]. Sequencing of the PCR products was performed by Macrogen (Daejeon, Korea). To search polymorphism in the promoter region of *Ghd7*, the 2 kb upstream region of *Ghd7* from Koshihikari and Baegilmi was sequenced using the customized sequencing service at Macrogen (Daejeon, Korea). Molecular markers to differentiate the three main polymorphisms in *Hd16*, *Hd1*, and *Ghd7* between Koshihikari and Baegilmi were developed (Supplementary Table S1) and used to screen 295 Korean rice cultivars released in 1979–2017 by NICS, Rural Development Administration.

## 3. Results

### 3.1. Days to Heading and Grain Filling Rate of Baegilmi in Comparison with Koshihikari

**Figure 1:**
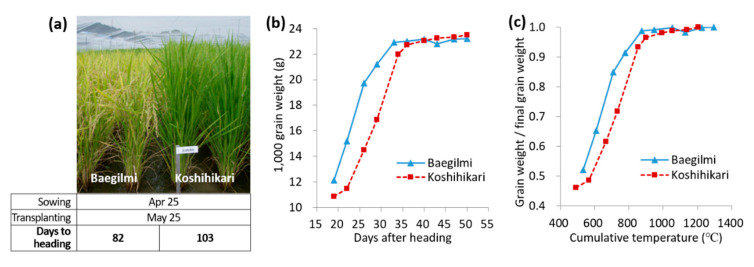
Maturity of Baegilmi in comparison with Koshihikari. (**a**) Representative phenotype of Baegilmi and Koshihikari in the field at 112 days after sowing (15 August). Days to heading was determined as the number of days from sowing to heading; (**b**) Changes in grain weight during 19–50 days after heading; (**c**) Changes in the proportion of grain weight to the final grain weight according to the cumulative temperature after heading.

The DH of Baegilmi under the optimum planting (i.e., sowing and transplanting on 25 April and 25 May, respectively, in Suwon, Korea) was 82 days, which was 21 days earlier than that of Koshihikari (Figure 1a). To compare the rate of grain filling of Baegilmi and Koshihikari, the grain weight change was monitored by measuring 1000 grain weight (TGW) every 3–4 days from 19 days after heading (DAH) to 50 DAH (Figure 1b). While the TGW of Baegilmi increased faster than that of Koshihikari during the early grain filling stage, reaching 19.7 g at 26 DAH (14.5 g in Koshihikari), the TGW of Koshihikari increased faster in the late grain filling stage, reaching 23.1g at 40 DAH which was similar to Baegilmi (23.2 g). A similar pattern was observed when the changes in grain weight were monitored as the ratio of grain weight to the final grain weight according to the cumulative temperature after heading (Figure 1c). Our results indicated that Baegilmi undergoes rapid grain weight increase in the early stage of grain filling as well as early vegetative-to-reproductive transition compared to Koshihikari.

### 3.2. Mapping of qDH3, qDH6, and qDH7 for Days to Heading

The Koshihikari × Baegilmi RIL population (*n* = 142) was used to map QTLs for DH. A GBS experiment detected 893 SNPs segregating in the mapping population. After removing SNPs with low genotyping quality and overlapping genetic positions, a linkage map spanning a total length of 1293 cM was constructed with the 128 selected SNPs (Supplementary Figure S1). The average number of markers per chromosome was 10.7, ranging from six on chromosome 3 to 17 on chromosome 6. The average interval between two adjacent markers was 11.1 cM.

**Figure 2:**
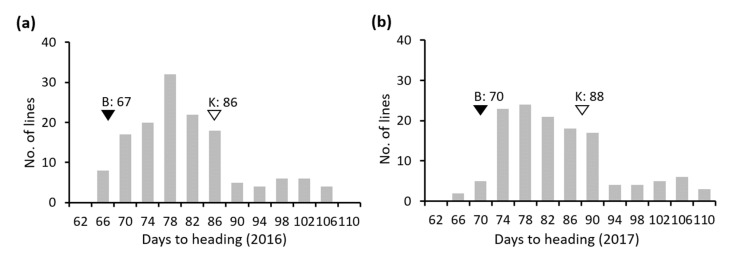
Frequency distribution of days to heading in the Koshihikari × Baegilmi RIL population evaluated in 2016 (**a**) and 2017 (**b**). The values of Baegilmi (B) and Koshihikari (K) are indicated above filled and unfilled triangles, respectively.

**Figure 3:**
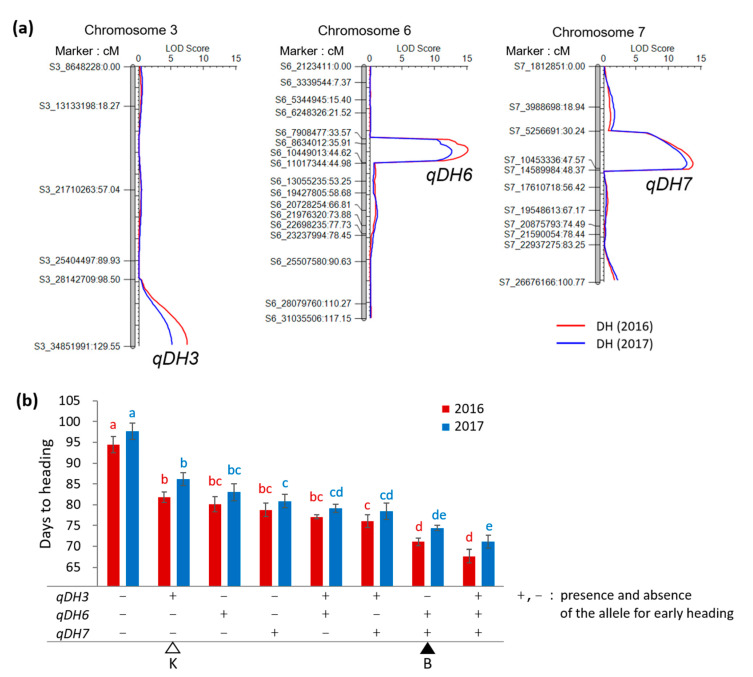
QTLs for days to heading from the Koshihikari × Baegilmi RIL population. (**a**) Mapping of *qDH3*, *qDH6*, and *qDH7* on chromosomes 3, 6, and 7, respectively. Days to heading of the RILs observed in 2016 and 2017 were used for mapping. The number after the letter ‘S’ in the marker name indicates the chromosome number followed by the physical position according to the IRGSP-1.0 reference; (**b**) Days to heading of the RILs with different allelic combinations of *qDH3*, *qDH6*, and *qDH7*. The markers S3_34851991, S6_8634012, and S7_10453336 were used to represent *qDH3*, *qDH6*, and *qDH7*, respectively. + and − indicate the presence and absence of the allele for early heading, respectively. The genotypes of Baegilmi (B) and Koshihikari (K) are indicated by filled and unfilled triangles, respectively. Different letters above the bars indicate significant difference according to the Duncan’s multiple range test at *p* < 0.05. Error bars indicate standard errors.

The Koshihikari × Baegilmi RILs showed continuous DH variation (64–105 days in 2016 and 64–107 days in 2017) with positively skewed distribution (Figure 2a,b). Baegilmi headed 19 and 18 days earlier than Koshihikari in 2016 and 2017, respectively. We detected three major QTLs for DH designated *qDH3*, *qDH6*, and *qDH7* on chromosomes 3, 6, and 7, respectively, in both 2016 and 2017 (Figure 3a, Table 1). At *qDH3*, Koshihikari contributed the allele for early heading with the additive effects of −3.1–−3.4 days and the LOD scores of 5.1–7.5, explaining 8.4–10.3% of the DH variation. On the other hand, Baegilmi contributed the allele for early heading at *qDH6* and *qDH7* (additive effects of 5.2–5.4 days and 5.0–5.3 days, respectively), which showed higher LOD scores (12.6–15.1 and 12.7–13.7, respectively) explaining higher levels of the DH variation (24.7–26.2% and 22.8–25.2%, respectively).

To analyze the main effects of the three QTLs and their two-way and three-way interactions, 2 × 2 × 2 factorial ANOVAs were conducted for DH of the Koshihikari × Baegilmi RILs (Table 2). The main effects of *qDH3*, *qDH6*, and *qDH7* were highly significant (3.8 × 10^−15^ < *p* < 2.6 × 10^−5^) in both 2016 and 2017. On average, the RILs carrying the Koshihikari alleles at *qDH3* headed 5.1–5.2 days earlier than those carrying the Baegilmi alleles. On the other hand, the RILs carrying the Baegilmi alleles at *qDH6* or *qDH7* headed 9.5–9.9 or 10.7–11.4 days earlier than those with the Koshihikari alleles, respectively. The RILs with different allele combinations of *qDH3*, *qDH6*, and *qDH7* showed that accumulating two or more alleles for early heading of theses QTLs can shorten DH effectively, with the pyramiding effect of *qDH6* and *qDH7* being greater than that of *qDH3* (Figure 3b).

**Figure 4:**
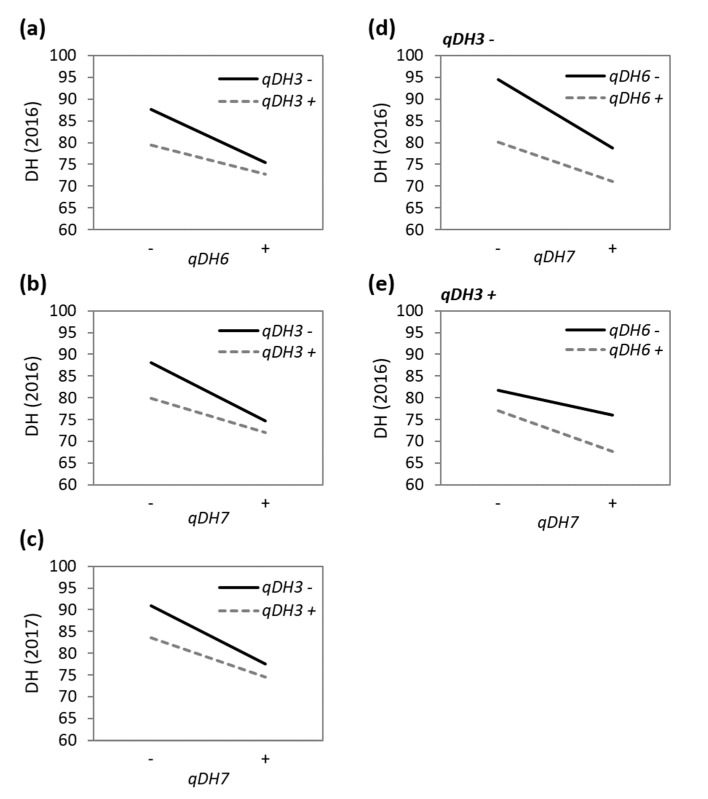
Interactions among the three QTLs for days to heading. (**a**) *qDH3* × *qDH6* interaction in 2016; (**b**) *qDH3* × *qDH7* interaction in 2016; (**c**) *qDH3* × *qDH7* interaction in 2017; (**d**) *qDH6* × *qDH7* interaction under the absence of the allele for early heading at *qDH3* in 2016; (**e**) *qDH6* × *qDH7* under the presence of the allele for early heading at *qDH3* in 2016. Only the significant (*p* < 0.05) interactions are plotted (see Table 2).

The two-way and three-way interactions among *qDH3*, *qDH6*, and *qDH7* were marginally significant (0.02 < *p* < 0.05) and explained little phenotypic variation (1.3–1.7%) (Table 2). The *qDH3 × qDH6* interaction was significant only in 2016, where the effect of *qDH6* accelerating heading was greater under the absence of the allele for early heading at *qDH3* (Figure 4a). Similarly, the effect of *qDH7* accelerating heading was greater under the absence of the allele for early heading at *qDH3*, and this was significant in both 2016 and 2017 (Figure 4b,c). The *qDH3 × qDH6 × qDH7* interaction was significant only in 2016. Under the absence of the allele for early heading at *qDH3*, the *qDH6 × qDH7* interaction pattern was similar to the *qDH3 × qDH6* and *qDH3 × qDH7* interactions (Figure 4d), i.e., the effect of one QTL accelerating heading was greater under the absence of the allele for early heading at the other QTL. However, under the presence of the allele for early heading at *qDH3*, the *qDH6 × qDH7* interaction pattern was reversed (Figure 4e), i.e., the effect of one QTL accelerating heading was greater under the presence of the allele for early heading at the other QTL.

### 3.3. Hd16, Hd1, and Ghd7 Underlying the Days to Heading QTLs

As the three QTLs for DH encompass previously isolated heading date genes—*Hd16* (*Os03g0793500*) at *qDH3*, *Hd1* (*Os06g0275000*) at *qDH6*, and *Ghd7* (*Os07g0261200*) at *qDH7* [7,10,23]—we sequenced the coding regions of the three genes from Koshihikari and Baegilmi to search sequence polymorphisms.

**Figure 5:**
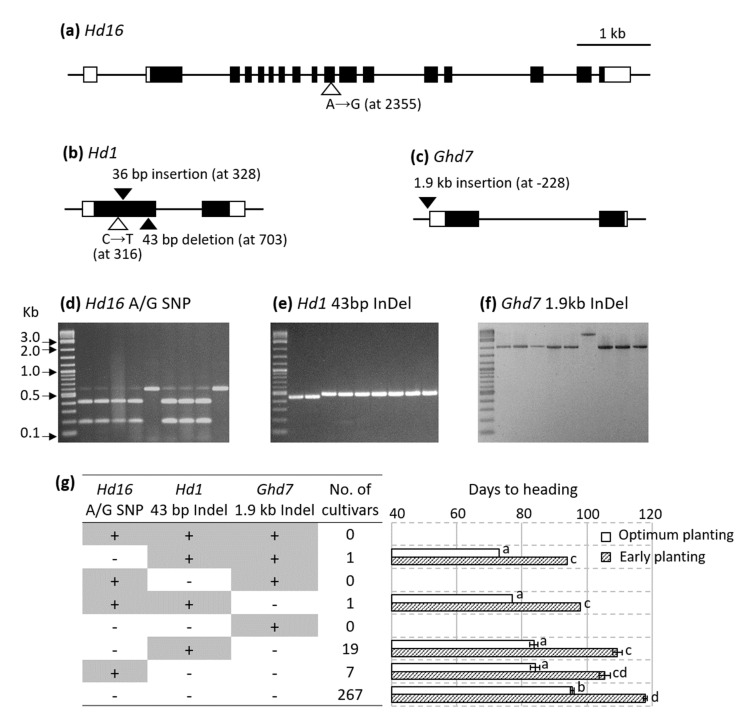
Sequence polymorphisms of *Hd16* (*Os03g0793500*), *Hd1* (*Os06g0275000*), and *Ghd7* (*Os07g0261200*) in Baegilmi relative to Koshihikari (**a**–**c**) and their genotyping among Korean rice cultivars (**d**–**g**). Coding region and untranslated region are depicted as filled and unfilled squares, respectively, while introns are depicted as black lines (**a**–**c**). Unfilled and filled triangles indicate SNP and insertion/deletion of Baegilmi in comparison with Koshihikari, respectively (**a**–**c**). The *Hd16* A SNP (Koshihikari allele) and the G SNP (Baegilmi allele) are visualized as intact (579 bp) and digested (393 bp + 186 bp) bands, respectively (**d**). The *Hd1* 43 bp deletion (Baegilmi allele) is visualized as a lower band (**e**). The *Ghd7* 1.9 kb insertion (Baegilmi allele) is visualized as an upper band (**f**). + and − indicate the presence and absence of the allele for early heading at each gene among 295 Korean rice cultivars, respectively, and the error bars in the bar graph indicate standard errors (**g**). Sowing and transplanting dates for optimum planting were 9 May and 1 June, respectively in 2018, and those for early planting were 10 April and 9 May, respectively in 2019, in Wanju, Korea. Different letters next to the bars indicate significant difference (*p* < 0.05) according to the Duncan’s multiple range test in each year.

It was previously shown that relative to the functional *Hd16* allele of Nipponbare, Koshihikari carries a G-to-A (alanine-to-threonine) mutation in the 10th exon of *Hd16* that is responsible for early heading under long day [23]. Sequence analysis of the 10th exon of *Hd16* revealed that the sequence of Baegilmi is identical to Nipponbare (Figure 5a). This was consistent with our QTL analysis where Koshihikari provided the allele for early heading and Baegilmi provided the allele for late heading at *qDH3* (Table 1), suggesting *Hd16* as a strong candidate gene for *qDH3*.

Sequence analysis of the *Hd1* coding region revealed that Koshihikari carries the functional *Hd1* allele identical to Nipponbare as previously reported [22], while Baegilmi carries a non-functional allele with three sequence polymorphisms relative to Koshihikari—a C-to-T SNP, a 36-bp insertion, and a 43-bp deletion in the first exon of *Hd1* (Figure 5b). The *Hd1* polymorphisms in Baegilmi are identical to those reported in HS66 (GeneBank ID AB041841), the γ adiation mutant of the Japanese cultivar Ginbouzu [7].

While the sequence analysis found no polymorphism in the coding region of *Ghd7* between Koshihikari and Baegilmi, we identified a 1901 bp insertion at the −228 bp position from the start codon of *Ghd7* in Baegilmi (Figure 5c). The position of the 1901 bp insertion and its sequence were identical to those of the putative retrotransposon inserted in the promoter region of *Ghd7* reported in the Japanese cultivar Sorachi (GenBank ID LC472532) [24].

### 3.4. Allelic Composition of Hd16, Hd1, and Ghd7 among Commercial Rice Cultivars

To study the allelic composition of the three DH genes in commercial rice cultivars, we screened 295 Korean rice cultivars released in 1979–2017 using the molecular markers designed to genotype the sequence polymorphisms in *Hd16* (A/G SNP), *Hd1* (43 bp Indel), and *Ghd7* (1.9 kb Indel) (Supplementary Table S1, Figure 5d–f). Of the eight (2 × 2 × 2) possible allelic combinations, five were observed among the 295 cultivars (Supplementary Table S2, Figure 5g). The majority (>90%) carried the alleles for late heading for all three polymorphisms, namely, the *Hd16* G SNP, the *Hd1* 43-bp insertion, and the *Ghd7* 1.9-kb deletion. Among 26 cultivars carrying an allele for early heading in one of three DH genes, 19 carried the *Hd1* 43-bp deletion and seven carried the *Hd16* A SNP. Only two out of the 295 Korean rice cultivars carried alleles for early heading in two of the three DH genes—Jopum carried the alleles for early heading at *Hd16* and *Hd1*, while Baegilmi carried the alleles for early heading at *Hd1* and *Ghd7*. None of the 295 cultivars carried the alleles for early heading at all three genes. The average DH of the five cultivar groups according to the allelic combinations of *Hd16* (A/G SNP), *Hd1* (43 bp Indel), and *Ghd7* (1.9 kb Indel) indicated that pyramiding the alleles for early heading at the three genes would accelerate DH effectively in the genetic background of commercial Korean rice cultivars (Figure 5g).

## 4. Discussion

Baegilmi is an extremely early heading Korean rice cultivar that can be incorporated in diverse double cropping systems to improve land use efficiency [14]. To genetically dissect the early heading characteristics of Baegilmi, we used a RIL population derived from the cross between Koshihikari and Baegilmi and identified the chromosomal locations harboring the genes controlling DH. We identified three major QTLs for DH, of which the allele for early heading was contributed by Baegilmi at *qDH6* and *qDH7*, and by Koshihikari at *qDH3*. As pyramiding of the alleles for early heading at each QTL was effective in accelerating heading, the Koshihikari × Baegilmi RIL population provides useful breeding lines with different allelic combinations of the three QTLs conferring varying DH, ranging from 71 days to 98 days under the natural long day condition in Wanju (36° N), Korea (Figure 3b). Therefore, the RIL population would be useful for selecting promising breeding lines potentially inheriting the good eating quality of Koshihikari with various heading dates that can be adapted in different environments and cropping patterns. To support this idea, we are currently evaluating the RILs with different allelic combinations of the three DH QTLs in terms of important agronomic traits such as yield performance and grain quality.

All three DH QTLs identified in this study harbor previously cloned heading date genes, i.e., *Hd1* [7], *Ghd7* [10], and *Hd16* [23] at *qDH6*, *qDH7*, and *qDH3*, respectively. *Hd1*, the ortholog of Arabidopsis *CO*, is the first heading date gene cloned in rice [7]. While *Hd1* accelerates heading under short day by upregulating *Hd3a*, it downregulates *Hd3a* under long day and represses heading [6,7,8,25]. Due to this dual function of *Hd1* depending on daylength, nonfunctional *hd1* alleles confer early heading phenotype under long day as the loss-of-function of *Hd1* de-represses *Hd3a* and promotes heading [26,27]. The nonfunctional *hd1* alleles have played an important role in expanding rice cultivation in high latitude areas with natural long day conditions where early heading is critical for rice plants to mature before the cold winter [27]. *Hd1* sequencing revealed that Baegimli carries a nonfunctional *hd1* allele due to the 43 bp deletion in exon 1 causing a frameshift (Figure 5b). The 43 bp deletion in *Hd1* was first reported in the γ ray mutant cultivar HS66 [7], and has been observed in many rice cultivars grown in high latitude areas such as Jilin (44° N) and Liaoning (41° N) of China [28] and Italy (35–47° N) [27]. Among the 295 Korean rice cultivars screened in this study, 21 including Baegilmi carried the nonfunctional *hd1* allele due to the 43 bp deletion and showed earlier heading phenotype compared to those without the 43 bp deletion (Figure 5g). As there are many other nonfunctional *hd1* alleles arising from frameshift or premature stop codon at different genic positions [22,26,27,28], further work is underway to define additional allelic variation in *Hd1* among the Korean rice cultivars.

Similar to *Hd1*, *Ghd7* encoding a CCT (CO, CO-LIKE, and TIMING OF CAB1) domain protein delays heading under long day by downregulating *Hd3a* [10]. In addition, similar to nonfunctional *hd1*, nonfunctional *ghd7* alleles such as *ghd7-0* (full gene deletion) and *ghd7-0a* (premature stop codon) accelerate heading under long day and contribute to the adaption of rice in high latitude areas [10,28]. Relative to Koshihikari, Baegilmi has a 1901 bp insertion in the promoter region (−228 bp from the start codon) of *Ghd7* (Figure 5c). The 1901 bp insertion is identical to that reported in *Ghd7* from a Japanese cultivar Sorachi (GenBank ID LC472533) with 449 bp long terminal repeats at each end and 5 bp (AGGTA) target site duplication (Fujino and Yamanouch 2020). This allele has been mainly found in rice cultivars bred in Hokkaido (42–45° N) of Japan, providing an important genetic source for rice adaption in high latitude [24,29]. Among the 295 Korean rice cultivars screened in this study, Baegilmi was the only cultivar carrying the loss-of-function *ghd7* allele due to the 1901 bp retrotransposon insertion (Figure 5g), suggesting that this allele would provide a valuable genetic source to accelerate heading in breeding programs. Interestingly, the same retrotransposon insertion has been also found in exon 2 of *Hd1* from a Taiwanese landrace Muteka (GenBank ID KR230393), creating a loss-of-function *hd1* allele [30]. This illustrates that transposable elements can play important roles in functionally diversifying the heading date genes.

*Hd16* encoding casein kinase I delays heading under long day by activating *Ghd7* through phosphorylation, and the missense mutation in exon 10 of *Hd16* from Koshihikari decreases the kinase activity of Hd16, thus accelerating heading under long day [23]. The screening of over 300 worldwide rice cultivars and 30 wild rice accessions revealed that the Koshihikari-type *Hd16* allele is found only in Japanese *japonica* cultivars [23]. Among the 295 Korean rice cultivars, only eight (i.e., Manan, Boseok, Jinkwang, Pungmi, Pungmi 1, Cheonga, Jungsaenggold, and Jopum) carried the Koshihikari-type *Hd16* and showed earlier heading phenotype compared to those carrying the Nipponbare-type *Hd16* (Figure 5g). These cultivars would provide useful genetic sources for developing early heading rice cultivars.

We previously reported that Baegilmi was developed by chemically mutagenizing Koshihikari [14]. However, it is extremely unlikely that the EMS mutagenesis would have created the above-mentioned sequence polymorphisms in *Hd1*, *Ghd7*, and *Hd16* that had been reported previously. This indicates that in the initial stages of breeding Baegilmi, the Koshihikari seed stock used for mutagenesis might have been contaminated during seed handling (e.g., threshing) with a different germplasm which may carry all or some of the previously known alleles, i.e., the HS66-type *Hd1*, the Sorachi-type *Ghd7*, and the Nipponbare-type *Hd16*. We currently do not know the exact source of the potential contamination. Although lacking a clear parentage, the practical value of Baegilmi as a cultivar is unaffected because Baegilmi carries a unique allelic composition of the three heading date genes and exhibits the second earliest heading date among the 295 Korean rice cultivars released in 1979–2017 [31]. Our study also suggests that developing cultivars with earlier heading would be possible by pyramiding the alleles for early heading at *Hd1*, *Ghd7*, and *Hd16*, as none of the 295 cultivars screened in this study carried the alleles for early heading at all three genes.

## 5. Conclusions

Baegilmi is a *japonica* rice cultivar with extremely early heading that is useful for diversifying cropping patterns in Korea. From the Koshihikari × Baegilmi RIL population, we detected three QTLs for days to heading, *qDH3*, *qDH6*, and *qDH7*. Different allelic combinations of the three QTLs in the RIL population provided breeding lines that can be useful for developing high quality *japonica* rice cultivars inheriting Koshihikari’s high eating quality with varying DH. Screening Korean rice cultivars for the allelic compositions of *Hd16* (A/G SNP), *Hd1* (43 bp Indel), and *Ghd7* (1.9 kb Indel) underlying *qDH3*, *qDH6*, and *qDH7*, respectively, showed that few cultivars carry one or more alleles for early heading at the three genes, suggesting that DH can be further accelerated and fine-tuned in breeding programs by using the allelic variations in *Hd16*, *Hd1*, and *Ghd7*.

## Figures and Tables

**Table 1:** Quantitative trait loci (QTLs) for days to heading from the Koshihikari × Baegilmi recombinant inbred line (RIL) population.

QTL	Chr ^a^	Flanking Markers ^b^		Year	Peak (cM)	LOD ^c^	PVE ^d^ (%)	Add ^e^
		Left	Right					
*qDH3*	3	S3_28142709	S3_34851991	2016	129	7.5	10.3	−3.4
				2017	129	5.1	8.4	−3.1
*qDH6*	6	S6_8634012	S6_10449013	2016	39	15.1	24.7	5.2
				2017	40	12.6	26.2	5.4
*qDH7*	7	S7_5256691	S7_10453336	2016	45	13.7	22.8	5.0
				2017	46	12.7	25.2	5.3

^a^ Chromosome number. ^b^ The number after the letter ‘S’ in the marker name indicates the chromosome number, followed by the physical location of SNP according to the IRGSP-1.0 reference. ^c^ Logarithm of the odds. ^d^ Phenotypic variation explained (%). ^e^ Additive effect of QTL estimated at the peak position. Positive additive effect indicates that the trait value is increased by the Koshihikari allele.

**Table 2:** Three-way ANOVAs of *qDH3*, *qDH6*, and *qDH7* for days to heading.

Year	Three-Way ANOVA					Mean DH ^c^	
	Effect ^a^		*F* Value	*p* Value	PVE ^b^	K	B
2016	Main	*qDH3*	24.5	2.5 × 10^−6^	7.6	76.4	81.6
		*qDH6*	63.7	1.2 × 10^−12^	19.8	83.4	73.9
		*qDH7*	82.3	3.8 × 10^−15^	25.5	84.3	73.6
	Interaction	*qDH3* × *qDH6*	4.1	0.0453	1.3		
		*qDH6* × *qDH7*	0.5	ns	-		
		*qDH3* × *qDH7*	4.8	0.0301	1.5		
		*qDH3* × *qDH6* × *qDH7*	5.5	0.0206	1.7		
2017	Main	*qDH3*	19.4	2.6 × 10^−5^	6.6	79.6	84.7
		*qDH6*	54.9	3.3 × 10^−11^	18.8	86.9	76.9
		*qDH7*	73.4	9.7 × 10^−14^	25.2	87.7	76.3
	Interaction	*qDH3* × *qDH6*	2.0	ns	-		
		*qDH6* × *qDH7*	2.8	ns	-		
		*qDH3* × *qDH7*	4.2	0.0433	1.4		
		*qDH3* × *qDH6* × *qDH7*	3.1	ns	-		

^a^*qDH3*, *qDH6*, and *qDH7* were represented by the markers S3_34851991, S6_8634012, and S7_10453336, respectively. ^b^ Phenotypic variation explained (%) is shown only for the significant (*p* < 0.05) effects. ^c^ Mean days to heading of the RILs carrying the Koshihikari (K) allele and the Baegilmi (B) allele at each of the three QTL.

## Supplementary Materials

The following are available online at https://www.mdpi.com/2073-4425/11/9/957/s1, Supplementary Figure S1: Linkage map from the Koshihikari × Baegilmi RIL population (*n* = 142) using 128 SNP markers, Supplementary Table S1: Molecular markers designed to genotype sequence polymorphisms in *Hd16*, *Hd1*, and *Ghd7*, Supplementary Table S2: Allelic distributions of the polymorphisms in *Hd16*, *Hd1*, and *Ghd7* in 295 Korean commercial rice cultivars.
