# Supplementary material for: Characterization of QTLs and Candidate Genes for Days to Heading in Rice Recombinant Inbred Lines
Source: Genes (Basel). 2020 Aug 19;11(9):957. doi: 10.3390/genes11090957 (PMC7565938; doi:10.3390/genes11090957)
Supplement: Supplementary file 1 [file genes-11-00957-s001.pdf]

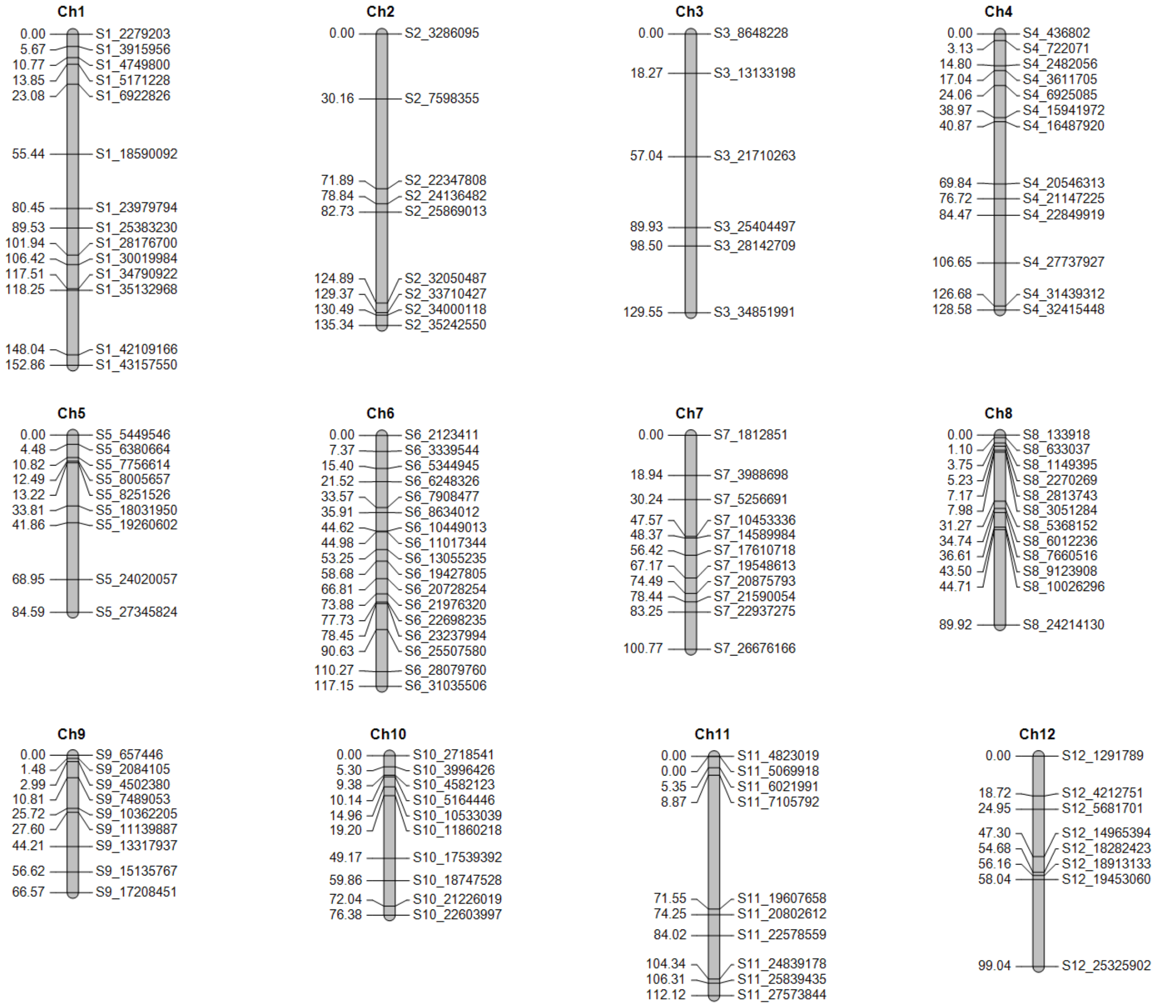

Supplementary Figure S1. Linkage map from the Koshihikari × Baegilmi RIL population (*n*=142) using 128 SNP markers. The numbers on the left of each chromosome indicate genetic distances (cM), and those on the right indicate the names of the SNP markers. The number after the letter ‘S’ in the marker name indicates the chromosome number followed by the physical position according to the IRGSP-1.0 reference.

Supplementary Table S1. Molecular markers designed to genotype sequence polymorphisms in *Hd16*, *Hd1*, and *Ghd7*.

Marker	Primer sequence (5' to 3') [a]	Tm [b] (°C)	Ext [c] (sec)	RE [d]	Band pattern (bp) [e]	
					K	B
Hd16 A/G SNP	F: GCAGCATCCAGGTGGAAAGAAGCA	68	60	*NheI*	579	393&186
	R: TGGGCCTTGAAGCAGGCCCTT					
Hd1 43 bp InDel	F: TCGAAAACAACCAAGATCGG	58	30	-	491	448
	R: TCGGTTCCATTTAATCAGCCT					
Ghd7 1.9 kb Indel	F: CGGCCGGATCAGGATTATTG	60	240	-	1,998	3,899
	R: CCGTCAGGGACTCAAAAGAG					

[a] Forward (F) and reverse (R) primer sequences; [b] Primer melting temperature; [c] Extension time; [d] Restriction enzyme used to digest the PCR product; [e] DNA band sizes of the Koshihikari (K) and Baegilmi (B) alleles on agarose gel.

Note: PCRs were performed with the initial denaturation at 94°C for 5 min, 35 cycles of denaturation at 94°C for 30 s, annealing at the relevant Tm for 30s, extension at 72°C for the relevant extension time (Ext), followed by the final extension at 72°C for 10 min. For the *Hd16* A/G SNP, PCR was conducted by combining the annealing and extension steps at 68°C, followed by the *NheI* digestion of the PCR product.

Supplementary Table S2. Allelic distributions of the polymorphisms in *Hd16*, *Hd1*, and *Ghd7* in 295 Korean commercial rice cultivars.

Cultivar	*Hd16*<br>A/G SNP	*Hd1*<br>43 bp indel	*Ghd7*<br>1.9 kb Indel	DH (2018)	DH (2019)
Baegilmi	-	+	+	73	94
Jopum	+	+	-	77	98
Asemi	-	+	-	85	109
Danpyeng	-	+	-	83	111
Hanseol	-	+	-	78	98
Heukjinjubyeo	-	+	-	81	98
Hwangkeumbora	-	+	-	88	105
Hwawang	-	+	-	85	108
Jeogjinju	-	+	-	81	103
Jinbuchalbyeo	-	+	-	81	101
Jinmibyeo	-	+	-	88	111
Jinseolchal	-	+	-	77	94
Jogwang	-	+	-	82	104
Josaengheugchal	-	+	-	86	110
Joun	-	+	-	79	98
Jungsan	-	+	-	88	110
Manchu	-	+	-	80	107
Manho	-	+	-	82	104
Mogyang	-	+	-	105	129
Obongbyeo	-	+	-	84	101
Ondami	-	+	-	85	107
BoSeog	+	-	-	78	104
Cheonga	+	-	-	85	112
Jinkwang	+	-	-	81	106
Jungsaenggold	+	-	-	87	115
Mananbyeo	+	-	-	85	108
Pungmi	+	-	-	84	110
Pungmi1	+	-	-	86	112
Anbaek	-	-	-	101	124
Andabyeo	-	-	-	95	117
Anmi	-	-	-	101	123
Aranghyangchalbyeo	-	-	-	99	124
Areumbyeo	-	-	-	95	118
Aromi	-	-	-	107	124
Asemi1ho	-	-	-	83	108
Baegjinju	-	-	-	105	128
Baegjinju1ho	-	-	-	105	130
Baegokchal	-	-	-	104	128
Baegseolchal	-	-	-	96	121
Bodrami	-	-	-	99	120
Boramchal	-	-	-	97	119
Boramchan	-	-	-	104	126
Borami	-	-	-	101	120
Boseogchal	-	-	-	95	117
Boseogheugchal	-	-	-	99	123
Cheongan	-	-	-	96	115
Cheongbaekchal	-	-	-	78	99
Cheongcheongbyeo	-	-	-	99	121
Cheongcheongjinmi	-	-	-	100	120
Cheongdam	-	-	-	94	119
Cheonghaejinmi	-	-	-	104	124
Cheongho	-	-	-	100	120
Cheonghyangheukmi	-	-	-	104	129
Cheongnam	-	-	-	95	119
Cheongpum	-	-	-	91	120
Cheongun	-	-	-	96	124
Cheongwoo	-	-	-	131	137
Chilbo	-	-	-	102	123
Chindeul	-	-	-	104	128
Chinnong	-	-	-	106	128

Cultivar	*Hd16*<br>A/G SNP	*Hd1*<br>43 bp indel	*Ghd7*<br>1.9 kb Indel	DH (2018)	DH (2019)
CW92MR	-	-	-	83	107
Dabo	-	-	-	99	120
Dacheong	-	-	-	109	132
Daeanbyeo	-	-	-	99	121
Daebo	-	-	-	96	123
Daecheongbyeo	-	-	-	100	124
Daejinbyeo	-	-	-	101	123
Daepyeong	-	-	-	94	120
Daeripbyeo1	-	-	-	96	117
Daesanbyeo	-	-	-	104	127
Dami	-	-	-	102	124
Danmi	-	-	-	102	125
DASAN1HO	-	-	-	94	119
Dasan2	-	-	-	93	117
Dasanbyeo	-	-	-	94	115
Deuraechan	-	-	-	104	124
Dodamssal	-	-	-	96	120
Donganbyeo	-	-	-	100	124
Dongbo	-	-	-	95	119
Donghaejinmi	-	-	-	99	123
Dongjin1ho	-	-	-	102	123
Dongjin2	-	-	-	100	120
Dongjinbyeo	-	-	-	103	126
Dongjinchalbyeo	-	-	-	100	123
Dunnaebyeo	-	-	-	77	97
Gancheokbyeo	-	-	-	93	117
Gangbaek	-	-	-	94	121
Gangchan	-	-	-	101	120
Geonganghongmi	-	-	-	101	125
Geonyang2	-	-	-	101	125
Geonyangmi	-	-	-	101	123
Geuman	-	-	-	94	122
Geumgang1	-	-	-	103	126
Geumobyeo	-	-	-	83	105
Geumyoung	-	-	-	83	107
Geunnun	-	-	-	100	121
Gihobyeo	-	-	-	92	118
Goami	-	-	-	101	127
Goami2	-	-	-	105	125
Goami3	-	-	-	101	120
Goami4	-	-	-	102	123
Gopum	-	-	-	99	120
Goun	-	-	-	79	100
Gurubyeo	-	-	-	85	110
Gyehwabyeo	-	-	-	99	124
Haechanmulgyeol	-	-	-	99	120
Haedamssal	-	-	-	83	107
Haedeul	-	-	-	85	112
Haepum	-	-	-	102	123
Haepyeong	-	-	-	91	118
Haepyeongchal	-	-	-	93	116
Haiami	-	-	-	99	119
Hanam	-	-	-	102	124
Hanareum	-	-	-	101	120
Hanareum2	-	-	-	97	118
Hanareum3ho	-	-	-	96	117
Hanareum4	-	-	-	102	124
Hanareumchal	-	-	-	101	118
Handeul	-	-	-	88	111
Hangangchal1	-	-	-	98	120
Hangangchalbyeo	-	-	-	100	120
Hangaru	-	-	-	101	123
Hanmauem	-	-	-	102	121
Heaoreumi	-	-	-	94	116

Cultivar	*Hd16*<br>A/G SNP	*Hd1*<br>43 bp indel	*Ghd7*<br>1.9 kb Indel	DH (2018)	DH (2019)
Heughyang	-	-	-	106	132
Heugjinmi	-	-	-	100	121
Heugkwang	-	-	-	92	118
Heugnambyeo	-	-	-	99	122
Heugseol	-	-	-	100	118
Heugsujeong	-	-	-	102	125
Hoanbyeo	-	-	-	101	126
Hojin	-	-	-	101	125
HONGJINJU	-	-	-	97	119
Honong	-	-	-	106	126
Hopum	-	-	-	101	123
Hopyung	-	-	-	104	124
Huimangchan	-	-	-	101	123
Hwaan	-	-	-	96	119
Hwabong	-	-	-	94	120
Hwajinbyeo	-	-	-	95	120
Hwajungbyeo	-	-	-	92	118
Hwanambyeo	-	-	-	104	124
Hwanggeumnodeul	-	-	-	104	124
Hwangkeumnuri	-	-	-	108	126
Hwarang	-	-	-	104	125
Hwasambyeo	-	-	-	104	121
Hwaseonchalbyeo	-	-	-	92	116
Hwaseongbyeo	-	-	-	92	115
Hwasinbyeo	-	-	-	101	125
Hwayeongbyeo	-	-	-	96	120
Hyangmibyeo1	-	-	-	96	118
Hyangnambyeo	-	-	-	103	122
Hyeonpum	-	-	-	104	129
Ilmibyeo	-	-	-	103	124
Ilpumbyeo	-	-	-	104	125
Jangseongbyeo	-	-	-	101	120
Jannganbyeo	-	-	-	90	119
Jeogjinju2	-	-	-	103	126
Jeogjinjuchal	-	-	-	88	113
Jinbaek	-	-	-	110	129
Jinbo	-	-	-	97	120
Jinbubyeo	-	-	-	81	103
Jinbuolbyeo	-	-	-	69	89
Jinhan	-	-	-	79	99
Jinok	-	-	-	80	101
Jinpum	-	-	-	89	117
Jinsumi	-	-	-	104	122
Joami	-	-	-	83	107
Joan	-	-	-	79	103
Joeunheukmi	-	-	-	81	106
Joil	-	-	-	82	107
Jonong	-	-	-	86	112
Jopyeong	-	-	-	79	104
Joryeongbyeo	-	-	-	86	110
Juanbyeo	-	-	-	93	118
Junam	-	-	-	103	125
Junghwabyeo	-	-	-	81	103
Jungmo1006	-	-	-	101	119
Jungmo1024ho	-	-	-	83	108
Jungmo1032	-	-	-	74	101
Jungmo1034	-	-	-	94	121
Jungmo1043	-	-	-	85	107
Jungwonbyeo	-	-	-	100	121
Keumo3	-	-	-	81	102
Keumobyeo1	-	-	-	94	120
Keunpum	-	-	-	100	124
Keunseom	-	-	-	98	119
Kuemobyeo	-	-	-	92	122

Cultivar	*Hd16*<br>A/G SNP	*Hd1*<br>43 bp indel	*Ghd7*<br>1.9 kb Indel	DH (2018)	DH (2019)
Malgeumi	-	-	-	103	124
Manbaek	-	-	-	106	129
Mangeumbyeo	-	-	-	101	122
Manjong	-	-	-	95	116
Manmi	-	-	-	105	124
Manna	-	-	-	84	111
Manpung	-	-	-	91	116
Manwol	-	-	-	92	117
Migwang	-	-	-	94	121
Miho	-	-	-	107	127
Mihyangbyeo	-	-	-	99	120
Mimyeon	-	-	-	91	116
Mipum	-	-	-	109	131
Misiru	-	-	-	100	121
Misomi	-	-	-	94	123
Miwoo	-	-	-	109	125
Mogwoo	-	-	-	134	140
MY298BB	-	-	-	101	127
MY299BK	-	-	-	104	127
Naepungbyeo	-	-	-	85	110
Namcheonbyeo	-	-	-	95	117
Namil	-	-	-	88	109
Nampungbyeo	-	-	-	99	121
Nampyeongbyeo	-	-	-	103	126
Namwonbyeo	-	-	-	79	102
Nokwoo	-	-	-	110	131
Nokyang	-	-	-	101	121
Nonganbyeo	-	-	-	94	117
Nunbora	-	-	-	98	122
Nunkeunheugchal	-	-	-	83	109
Nunkeunheugchal1ho	-	-	-	82	107
Odae1ho	-	-	-	79	102
Odaebyeo	-	-	-	84	105
Onnuri	-	-	-	101	122
Palbangmi	-	-	-	97	119
Palgongbyeo	-	-	-	98	121
Pyeongwon	-	-	-	83	104
Saechilbo	-	-	-	99	122
Saegoami	-	-	-	98	123
Saegyewha	-	-	-	101	121
Saeilmi	-	-	-	101	123
Saeilpum	-	-	-	103	126
Saemimyeon	-	-	-	101	122
Saenuri	-	-	-	106	126
Saeodae	-	-	-	80	103
Saesangju	-	-	-	84	108
Saesin	-	-	-	109	129
Samcheonbyeo	-	-	-	82	103
Samdeog	-	-	-	94	119
Samgangbyeo	-	-	-	97	117
Samkwang	-	-	-	96	124
Samkwang1ho	-	-	-	91	117
Sampyeong	-	-	-	96	118
Sandeuljinmi	-	-	-	85	110
Sangbo	-	-	-	95	118
Sangjubyeo	-	-	-	83	107
Sangjuchalbyeo	-	-	-	85	109
Sangnambatbyeo	-	-	-	83	105
Sanhomi	-	-	-	82	105
Segyejinmi	-	-	-	96	116
Seoan1ho	-	-	-	97	120
Seoanbyeo	-	-	-	92	119
Seolbaek	-	-	-	79	103
Seolemi	-	-	-	83	109

Cultivar	*Hd16*<br>A/G SNP	*Hd1*<br>43 bp indel	*Ghd7*<br>1.9 kb Indel	DH (2018)	DH (2019)
Seolgaeng	-	-	-	104	126
Seolhyangchal	-	-	-	89	115
Seomyeong	-	-	-	102	124
Seongsan	-	-	-	84	109
Seonpum	-	-	-	100	124
Seopyeong	-	-	-	100	119
Shinbaeg	-	-	-	99	125
Shinpyeong	-	-	-	79	103
Sinbo	-	-	-	100	120
Sindongjin	-	-	-	101	123
Singil	-	-	-	99	118
Sinjinbaek	-	-	-	105	127
Sinseonchalbyeo	-	-	-	92	117
Sinunbong1	-	-	-	80	106
Sinunbongbyeo	-	-	-	79	103
Sobaegbyeo	-	-	-	77	98
Sobi	-	-	-	94	120
Sodami	-	-	-	105	126
Suan	-	-	-	98	121
Sujin	-	-	-	104	127
Sukwang	-	-	-	101	123
Surabyeo	-	-	-	91	118
Suryeojinmi	-	-	-	98	120
Taebaekbyeo	-	-	-	91	114
Taebong	-	-	-	79	101
Tamjinbyeo	-	-	-	106	127
Unbaekchal	-	-	-	80	103
Unbongbyeo	-	-	-	80	101
Undoobyeo	-	-	-	77	97
unilchal	-	-	-	81	106
Unjangbyeo	-	-	-	79	101
Unkwang	-	-	-	83	105
Unmi	-	-	-	81	105
Wolbaek	-	-	-	81	106
Yangjobyeo	-	-	-	100	121
Yechan	-	-	-	101	126
Yeongan	-	-	-	101	123
Yeongdeogbyeo	-	-	-	98	122
Yeongwoo	-	-	-	105	127
Yongmunbyeo	-	-	-	94	116
Youngbo	-	-	-	95	118
Younghojinmi	-	-	-	109	130
Youngjin	-	-	-	103	127

+ and – indicate the presence and absence of the allele for early heading at each gene among 295 Korean rice cultivars, respectively.

DH: days to heading (Sowing and transplanting dates for optimum planting were 9 May and 1 Jun, respectively in 2018, and those for early planting were 10 Apr and 9 May, respectively in 2019, in Wanju, Korea).
